# Analysis of the Biomass Composition of the Demosponge *Amphimedon queenslandica* on Heron Island Reef, Australia

**DOI:** 10.3390/md12063733

**Published:** 2014-06-23

**Authors:** Jabin R. Watson, Timothy C. R. Brennan, Bernard M. Degnan, Sandie M. Degnan, Jens O. Krömer

**Affiliations:** 1 School of Biological Science, University of Queensland, Brisbane, Queensland 4072, Australia; E-Mails: jabin.watson@uq.edu.au (J.R.W.); b.degnan@uq.edu.au (B.M.D.); s.degnan@uq.edu.au (S.M.D.); 2 Systems and Synthetic Biology Group, Australian Institute for Bioengineering and Nanotechnology, University of Queensland, Brisbane, Queensland 4072, Australia; E-Mail: t.brennan3@uq.edu.au; 3 Centre for Microbial Electrosynthesis (CEMES), Advanced Water Management Centre, University of Queensland, Brisbane, Queensland 4072, Australia

**Keywords:** Porifera, biochemical composition, *Amphimedon queenslandica*, amino acid analysis, FAME analysis, spongin

## Abstract

Marine sponges are a potential source of important pharmaceutical drugs, the commercialisation of which is restricted by the difficulties of obtaining a sufficient and regular supply of biomass. One way to optimize commercial cell lines for production is the in-depth characterization and target identification through genome scale metabolic modeling and flux analysis. By applying these tools to a sponge, we hope to gain insights into how biomass is formed. We chose *Amphimedon queenslandica* as it has an assembled and annotated genome, a prerequisite for genome scale modeling. The first stepping stone on the way to metabolic flux analysis in a sponge holobiont, is the characterization of its biomass composition. In this study we quantified the macromolecular composition and investigated the variation between and within sponges of a single population. We found lipids and protein to be the most abundant macromolecules, while carbohydrates were the most variable. We also analysed the composition and abundance of the fatty acids and amino acids, the important building blocks required to synthesise the abundant macromolecule types, lipids, and protein. These data complement the extensive genomic information available for *A. queenslandica* and lay the basis for genome scale modelling and flux analysis.

## 1. Introduction

Marine sponges and their associated bacteria are prolific producers of secondary metabolites that are an important source of bioactive compounds with pharmacological potential. More than 5000 compounds have been identified to date, including 355 new compounds reported in 2012 alone [1]. However, despite this enormous potential, approval for use as a therapeutic compound is scarce [2]. The lack of commercial development of sponge-derived compounds is attributed to both a biomass supply problem—no reliable method to culture either whole sponges or cells exists—and a lack of understanding of sponge-bacterial interactions. Together, these constraints limit the applicability of metabolic engineering approaches to microbial isolates or sponge cells for over-production of secondary metabolites. To move forward in the development of sponge-derived drugs requires extending the research activities beyond the current major focus on identifying secondary compounds and, to a limited extent, characterizing the biosynthetic pathways that produce these secondary metabolites [3]. We need to understand how the sponge holobiont (the animal and its resident microbes) is able to grow; understanding how the central metabolic pathways contribute to biomass production is crucial to overcoming the biomass supply problem.

The metabolic processes underlying sponge growth can be comprehensively investigated by the application of genome scale modeling and flux analyses [4]. An essential step of such analyses is characterization of the biochemical composition of the sponge holobiont, with a focus on the components that contribute significantly to overall biomass production [5]. Characterizing the biochemical composition allows the formation of a biomass equation, essential for genome scale modeling and flux analysis as it constrains hundreds of fluxes through the system [6]. The focus on secondary metabolic compounds has left the products of central metabolism comparatively overlooked, including analysis of the major cellular macromolecules—protein, lipid, carbohydrate, DNA, and RNA—and their constituent building blocks—amino acids, fatty acids, sugars and nucleotides. Although protein, lipid, and carbohydrate content has been quantitated in a number of marine sponges [7,8,9,10,11,12], a complete analysis of the main biomass components has not been reported.

In addition to quantifying the components that contribute to biomass formation, information about how variable the composition is across the population and within an individual sponge is important for guiding the design of experiments associated with flux analysis. Although sponges appear to be morphologically simple and homogenous, it has been shown that production of secondary metabolites can be controlled and restricted to specific areas of an individual [13]. This apparent heterogeneity may be related to physiological differences in the sponge itself or in the associated microbial community. Marine sponges typically host large and diverse communities of microbes [14], often in a symbiotic relationship [15]. These microbes have been shown to be the source of certain secondary metabolites [16], and also to interact with central metabolic pathways in the host sponge [17]. Thus the distribution of particular bacterial species and communities within an individual sponge has the potential to influence local metabolic activity. Nonetheless, subsampling the main biomass components within an individual sponge typically has not been performed, as most biochemical analyses performed in sponges have been restricted to a single sample from an individual or small number of individuals within a population [7,8,9,10,11,12]. Analysing multiple samples within individuals will give insights into the variation in the macromolecule composition within an individual sponge.

We report here the first analysis of the products of central metabolism of the marine demosponge, *Amphimedon queenslandica* from the Southern Great Barrier Reef. We have quantified the protein, lipid, carbohydrate, DNA and RNA content of this sponge. These analyses are supplemented with a detailed analysis of amino acid and fatty acid composition. By analysing multiple areas within multiple individuals within one *A. queenslandica* population we reveal marked variation within and between sponges for most of these macromolecular classes. These results, along with the extensive genomic resources available for this demosponge [18], provide a strong foundation for future detailed understanding of the metabolic pathways that contribute to biomass production.

## 2. Results

### 2.1. Displacement to Dry Weight Composition Conversion Factor

Metabolic flux analysis requires the ability to quantify the biomass involved in the turn-over of the substrate. In order to relate the amount of live sponge to the dry weight composition, we established a correlation between displacement volume of a sponge piece and its dry weight. The displacement volume and dry weight from 97 samples were used to estimate a conversion factor of 0.091 (std. dev. = 0.012) grams of dry sponge weight (gDW) per mL of displacement.

**Figure 1:**
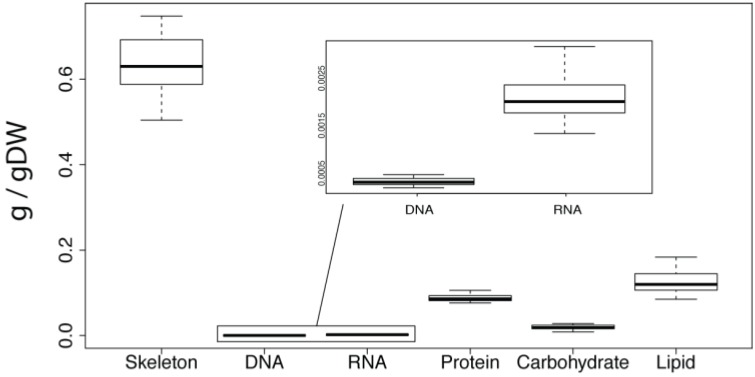
Mean skeleton and macromolecular composition of *A. queenslandica* calculated from all biomass samples summarised in Table 1. The box represents the upper and lower quartile, split by the mean value. The upper and lower whiskers denote the minimum and maximum values. Units are grams per gram of dry weight.

### 2.2. Overall Macromolecular Composition

For each biochemical analysis, we analysed five biopsies from several parts of four *A. queenslandica* individuals, all of which were collected from a single wild population in Shark Bay on Heron Island Reef, Australia (for detailed methods see Section 4). This sampling regime allowed us to document biochemical differences both within and between individual sponges. Analysis of mean values of the macro-components shows the skeleton (0.6342 g/gDW) to be the dominant component of the dry weight of *A. queenslandica* (Figure 1 and Table 1). The most abundant macromolecule type was lipid with 0.1251 g/gDW, followed by protein (0.0881 g/gDW), carbohydrate (0.0197 g/gDW), RNA (0.0021 g/gDW) and DNA (0.0003 g/gDW). The coefficient of variation revealed that carbohydrates were the most variable, followed by DNA, RNA, and lipids (Table 1). Protein and skeleton had the least overall variability (Table 1).

**Table 1:** Mean skeletal and macromolecular composition of *A. queenslandica* calculated from all biomass samples.

Macro-Component	Mean (g/gDW)	Standard Deviation	Standard Error	Coefficient of Variation (%)	*n*
Skeleton	0.6343	0.0647	0.0152	9.81	18
Lipid	0.1252	0.0251	0.0058	20.09	19
Protein	0.0881	0.0082	0.0018	9.31	20
Carbohydrate	0.0197	0.0055	0.0012	27.53	20
RNA	0.0021	0.0005	0.0001	22.24	20
DNA	0.0003	0.0001	0.00002	25.78	19

### 2.3. The Source of Variation in the Overall Composition

**Figure 2:**
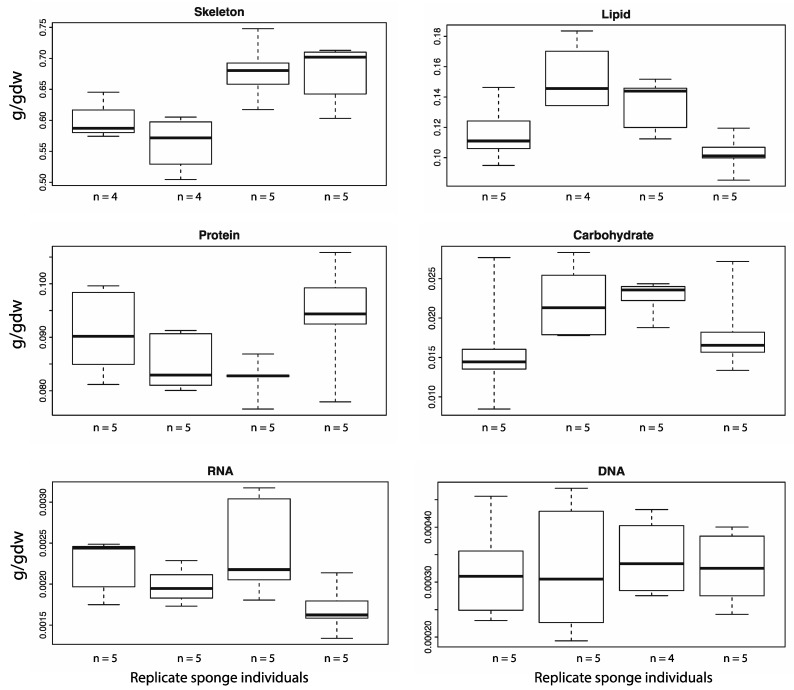
Variation of the composition of macro-components within and between individuals of *A. queenslandica.* Four individual sponges were sampled for each macromolecule. Each box and whisker plot represents samples collected from an individual sponge. The box represents the upper and lower quartile, split by the mean value. The upper and lower whiskers denote the minimum and maximum values. All of the components apart from DNA showed marked variation between and within individual sponges.

We used a linear mixed effects model to investigate how much of the variation observed in the mean values (Table 1, Coefficient of variation column) was attributable to the between-individual variation. Over half of the variation seen in the skeleton (58%) and lipids (54%) was due to differences between individuals. The significance of this relationship was tested using a *x*^2^ test. The variation of skeleton (*p* = 0.009) and lipids (*p* = 0.016) between individuals had significant effects on the overall variation of both macromolecule types (Table 2). Figure 2 shows the variation seen within each individual for the respective macro-components.

**Table 2:** The source and degree of variation.

Macro-Component	Percentage of Overall Variation Caused by between Individual Variation (%)	*x*^2^	*P*-value
Skeleton	58	6.691	0.009
Lipid	54	5.763	0.016
Protein	24	1.393	0.237
Carbohydrate	15	0.597	0.439
RNA	1	2.309	0.128
DNA	32	3.53 × 10^−8^	0.999

### 2.4. Fatty Acid and Sterol Analysis

The lipid extractions were analysed using a fatty acid methyl ester (FAME) analysis using gas chromatography-mass spectrometry (GC-MS). We were interested in the main components that contribute to forming sponge lipids. Compounds were considered essential if they were found in most of the 23 replicate pieces of sponge. Eleven fatty acid (FA) compounds were identified and confirmed with standards. Palmitic acid was the most abundant FA, contributing 98.14 μmol/gDW. Octadecanoic acid (62.21 μmol/gDW) and an unknown FA (UU4-FA, 71.749 in Table 3) were other significant FAs. Three additional compounds matched compounds in the NIST database but were not confirmed with a standard (Table 3), this included cholesterol. Based on the fragmentation patterns, eleven compounds were classified as unknown FAs and one as an unknown sterol, highlighting the diversity and complexity of the lipid metabolism in *A. queenslandica*. If the total area under all peaks in the chromatogram is assumed to represent the total lipid fraction, then the main FA and sterols reported here cover 82.5% of the lipid fraction.

**Table 3:** Main compounds that contribute to the lipid biomass.

Compound	Lipid Number	μmol/gDW	Standard Deviation
**Fatty Acids**			
Docosanoic acid FAME	C22:0	9.769	4.299
Eicosanoic acid FAME	C20:1	1.947	0.579
Erucic acid FAME	C22:1ω9	1.721	0.668
Heptadecanoic acid FAME	C17:0	0.922	0.234
Myristic acid FAME	C14:0	3.957	1.455
Nervonic acid FAME	C24:1ω9	10.090	3.451
Octadecanoic acid FAME	C18:0	62.210	19.095
Palmitic acid FAME	C16:0	98.140	22.426
Pentadecanoic acid FAME	C15:0	7.065	2.468
Tetracosanoic acid FAME	C24:0	6.769	3.546
Tricosanoic acid FAME	C23:0	1.147	0.323
11-Eicosenoic acid FAME *	C20:1ω9	5.342	1.737
U6-FA **		1.005	0.580
U7-FA **		5.973	3.845
U8-FA **		2.279	0.722
U10-FA **		3.288	3.014
UU2-FA **		7.732	2.805
UU3-FA **		5.669	1.897
UU4-FA **		71.749	17.820
UU7-FA **		0.690	0.604
UU8-FA **		4.215	2.155
UU9-FA **		29.843	10.551
UU11-FA **		4.460	1.846
UU1 ***		1.728	1.766
UU6 ***		9.617	4.268
**Sterols**			
Cholesterol *		4.706	2.477
Brassicasterol *		7.581	4.806
UU13-sterol **		6.264	3.375

* Compounds that did not match a compound in the standard mix but matched a compound in a database; ** interpreted from the fragmentation pattern; *** compounds that are neither FA nor sterols.

### 2.5. Amino Acid Analysis

We analysed the amino acid composition of 20 samples of complete sponge biomass (Table 4). Samples of biomass were lyophilized and the hydrolysis incubation time was optimized before quantifying the amino acids with high performance liquid chromatography (HPLC). We found glycine to be the most abundant amino acid with 4.582 mmol/gDW, followed by alanine (1.538 mmol/gDW). Due to the oxidation of asparagine and glutamine to aspartate and glutamate respectively during acid hydrolysis, a 50:50 abundance ratio was assumed between asparagine:aspartate and glutamine:glutamate. Tryptophan (0.089 mmol/gDW), tyrosine (0.157 mmol/gDW), and histidine (0.110 mmol/gDW) were the least abundant amino acids. Tryptophan and methionine are known to be degraded during the hydrolysis. While methionine was not detected tryptophan was measurable but *in vivo* concentrations may be higher than what was observed. Neither cysteine, nor cystine was measured with the methods used and thus not reported.

**Table 4:** Mean amino acid composition of complete sponge biomass.

Amino Acid	mmol/gDW	Standard Deviation (mmol/gDW)	Amino Acid Contribution (mol/mol)
ALA	1.538	0.565	0.097
ARG	0.694	0.258	0.044
ASP	0.841	0.312	0.053
ASN	0.841	0.312	0.053
GLN	0.767	0.287	0.048
GLU	0.767	0.287	0.048
GLY	4.582	1.702	0.289
HIS	0.110	0.042	0.007
ILE	0.402	0.149	0.025
LEU	0.597	0.222	0.038
LYS	0.688	0.268	0.043
PHE	0.369	0.137	0.023
PRO	1.050	0.387	0.066
SER	0.806	0.318	0.051
THR	0.826	0.304	0.052
TRP *	0.089	0.053	0.006
TYR	0.157	0.060	0.010
VAL	0.753	0.281	0.047

* Tryptophan is largely destroyed during the hydrolysis, so concentrations *in vivo* may be higher than observed.

The skeleton is the most abundant component in *A. queenslandica* and is comprised of inorganic silica spicules and a collagen based extracellular matrix. Sponges produce a specific collagen, called spongin [19]. As the skeleton contributes significantly to the overall sponge composition, we analysed the amino acid composition of the skeleton (Table 5). The hydrolysis incubation time was optimized before the analysis was performed on 20 samples. As above, a 50:50 ratio between aspartate and asparagine and between glutamine and glutamate was assumed. The most abundant amino acids in the skeleton were glycine with 4.248 mmol/gDW and alanine with 1.257 mmol/gDW. The least abundant amino acids were tyrosine (0.066 mmol/gDW) and histidine (0.034 mmol/gDW).

**Figure 3:**
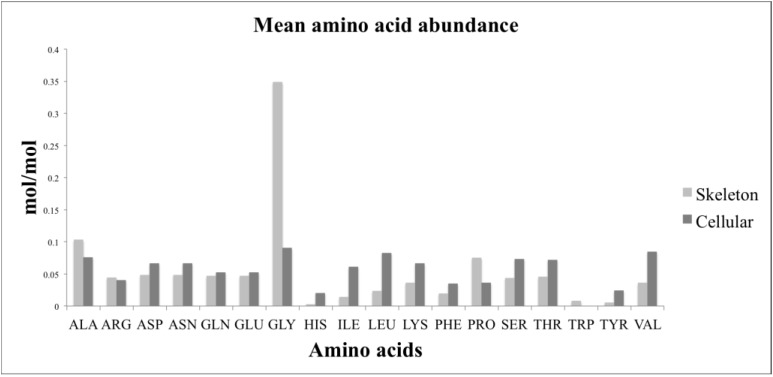
A comparison of the relative amino acid composition of the cellular biomass and the skeleton. The amino acid composition of the cellular components was calculated by subtracting the quantified amino acid values of the skeleton (Table 5) from the values for the complete sponge biomass (Table 4). The skeletal and estimated cellular amino acid values were then normalized by converting to percentages.

A comparison of the relative amino acid composition of the skeleton and the cellular component revealed that glycine was the most abundant amino acid for both components (Figure 3). The skeleton had relatively more glycine, proline, and alanine than the cellular fraction. The cellular fraction had a higher relative abundance of isoleucine, leucine, and valine than the skeleton. Histidine and tyrosine were the least abundant of the amino acids measured for both the skeleton and cellular components.

**Table 5:** Mean amino acid composition of the skeleton.

Amino Acid	mmol/gDW	Standard Deviation (mmol/gDW)	Amino Acid Contribution (mol/mol)
ALA	1.257	0.537	0.103
ARG	0.545	0.226	0.045
ASP	0.594	0.250	0.049
ASN	0.594	0.250	0.049
GLN	0.573	0.243	0.047
GLU	0.573	0.243	0.047
GLY	4.248	1.807	0.349
HIS	0.034	0.016	0.003
ILE	0.176	0.080	0.014
LEU	0.290	0.121	0.024
LYS	0.442	0.173	0.036
PHE	0.239	0.102	0.020
PRO	0.916	0.351	0.075
SER	0.534	0.211	0.044
THR	0.559	0.226	0.046
TRP	0.100	0.041	0.008
TYR	0.066	0.028	0.005
VAL	0.440	0.177	0.036

### 2.6. Nucleotide Composition

The molar contribution of nucleotides to the DNA and RNA composition was estimated using available genome and transcriptome data. The genomic GC content was estimated to be 31.14% using the publicly available genome [18]. The RNA GC content was estimated to be 39.76%, using an adult transcriptome [20]. These percentages were then converted into micromoles per gram of dry weight, using the molecular weight of a polymerized nucleotide (Table 6 and Table 7).

**Table 6:** Deoxyribonucleotide composition.

	MW (g/mol)	mol/ mol DNA	μmol/gDW
dAMP	313	0.3443	0.37
dCMP	289	0.1557	0.17
dGMP	329	0.1557	0.17
dTMP	304	0.3443	0.37

**Table 7:** Ribonucleotide composition.

	MW (g/mol)	mol/ mol DNA	μmol/gDW
AMP	313	0.3012	2.03
CMP	289	0.1988	1.34
GMP	329	0.1988	1.34
UMP	308	0.3012	2.03

## 3. Discussion

### 3.1. Macromolecule Composition

To gain insights into how sponges produce biomass, the complex metabolic processes must be investigated as a complete functioning system. The systems biology approach of genome scale metabolic modeling requires the biochemical composition of the organism to be known. The biochemical composition identifies the products of central metabolism that contribute to growth. With the products of central metabolism known, the synthesis pathways can be identified and the fluxes of metabolites modeled. As a first step towards genome scale modeling, we analysed the macromolecular abundance and composition of the marine sponge *A. queenslandica* and investigated the variation seen across the population and within individuals*.*

The skeleton was the dominant component of the biomass, contributing 63% of the dry weight. Lipids and protein were the most abundant macromolecules, while carbohydrates were the most variable (Table 1). Variation in the amount of skeleton and lipids between individual sponges had significant effects on the overall variability of the respective macro-components (Table 2). The apparent variation in the abundance of macromolecules within an individual (Figure 2) was not significant and had limited effect on the overall population variability. This implies that not only do the central metabolic pathways have an overall response to the individual’s microenvironment, but localised stimulus could also cause compositional differences across an individual. This highlights the need for compositional and metabolic studies to sample appropriately to ensure accurate representation.

Overall, the macromolecular composition of *A. queenslandica* in the current study fits within the ranges reported for protein, lipids, and carbohydrates for 71 tropical marine sponge species [7]. *A. queenslandica* notably contained more lipids than protein (Table 1), a characteristic that has been reported previously for two marine demosponges, *Aplysinia archeri* and *Plaktortis halichondroides* [7]. The high lipid content in *A. queenslandica* could indicate the accumulation of energy storage products in response to increased food availability and, thus, variably depending on food abundance. Alternatively, Elvin [8] found that, for *Haliclona permollis*, an increase in lipid content was associated with oogenesis and the formation of mesenchymal amoebocytes. Although *A.* *queenslandica* is reproductively active year round, larval production peaks in the warmer months [21]. It is not clear if oogenesis is the reason for the high lipid content as the sampling time of this study corresponds with the end of summer when reproductive output is starting to decline. In addition, *A. queenslandica* concentrates its oocytes and embryos in localised brood chambers [21], unlike many sponges, suggesting that these levels should vary markedly within an individual sponge.

In *A. queenslandica*, the cellular biomass accounts for less than half of the dry weight. We defined the skeleton as the solid, inorganic spicules and the flexible, organic collagen. The skeleton, being a product of cellular metabolism, is a considerable sink of metabolic resources, accounting for 64% of the total dry weight (Table 1). The amount of skeleton in *A. queenslandica* is within the ranges of both Antarctic [12] and tropical [7] sponges. The high skeletal requirement by all sponges, greater than 50% dry weight, is likely due to the lack of defined tissues, including connective tissues, that are required to support complex body plans. Without the significant support and framework that the skeleton provides, sponges would not be able to achieve the sizes observed across the phylum [22].

Although sponges do not have defined tissues, the distribution of cells in *A. queenslandica* does not appear to be even if DNA is used as a proxy for cell number. This could be in part due to the large size range of sponge cells, as smaller cells are able to occupy less space yet all cells contain the same amount of DNA. Cells typically contain more RNA than DNA [23], and this is also the case for *A. queenslandica*, in which RNA was more abundant than DNA.

### 3.2. The Variation in Macromolecule Composition

Metabolic networks are dynamic, plastic, and redundant [23]. These characteristics have evolved to give organisms the flexibility to rapidly adapt to variations in environment or external stimulus [23]. As the aim of this study is to gather baseline data for use in metabolic modeling, we sampled different individuals and took sub-samples within each individual to investigate the source of potential variation. This is important information that will help guide future work. There was marked variation both between and within individual sponges in the abundance of macromolecules (Figure 2).

The skeleton was the most abundant component, and the variation in between-individual abundance was shown to have a significant effect on the overall population variation. The skeleton is the structural support for the cellular biomass and it guides the irregular canal network through which water is pumped to supply food and oxygen [14]. The variability seen in the amount of skeleton is likely a result of *A. queenslandica*’s encrusting-lobate growth form [24], combined with the irregular substrate and different structural requirements of specific internal features, such as canals and brood chambers.

It was previously mentioned that the high lipid content could be due to the reproductive state of the population. The reproductive state would also explain the variability in the lipid content within an individual. *A. queenslandica* forms localised brood chambers where embryos are matured before release [25] and not all of the biopsies would have contained brood chambers.

The variation in macromolecule composition may underlie the metabolic responses of the sponge to an unpredictable and competitive environment. Thus, at the scale of within an individual, differences in microhabitat such as food abundance, predatory pressures and conflicts for space can influence the macromolecular composition. This is potentially exacerbated by the nature of the biochemical assays and the error associated with correcting for salt content. Our results reveal significant effects of between-individual variation and a lesser effect of within-individual variation. This indicates that future work on the composition or metabolic processes of *A. queenslandica* should maintain a minimum of four representative individuals, supplemented with at least some subsampling within each individual. Care should still be taken with experimental design as this dataset is representative of a single metabolic state, and assumptions relating these results to different seasonal or ontogenetic stages should be made with caution. These results highlight the importance of biomass composition analysis, rather than the use of literature values.

### 3.2. Fatty Acid and Sterol Composition

The lipidome consists of a mixture of fatty acids and sterols, of which sponges are renowned for their diversity and often unique compounds [26,27,28,29]. Despite this diversity among FA structures in sponges, there are no clear or reliable taxonomic trends [26,28]; this can in part be attributed to the microbial communities associated with sponges [28]. We did not want to focus on identifying each compound in the lipid fraction as this is not necessary for modeling the sponge holobiont and performing flux balance analysis. As the precursor molecule for all lipid synthesis is acetyl coenzyme A, flux balance analysis may be performed if the contribution of lipids towards the overall biomass is known. We identified what compounds matched our databases as their inclusion will increase the models accuracy. Unidentified compounds were classified as FAs, sterols or neither based on their fragmentation pattern.

The two most abundant FAs in *A. queenslandica* were palmitic acid and octadecanoic acid. Both of these FAs are common in nature and have been reported in sponges previously [30,31,32,33,34]. Palmitic acid is a precursor with chain elongation, desaturation and addition of side chain modifications possible [35]. Apart from pentadecanoic acid (15:0) and heptadecanoic acid (17:0), the remaining identified FAs are long chain FA. FA with carbon chain length 20 (eocosanoic acid), 22 (docosanoic acid) and 24 (tetracosanoic acid), along with desaturated FAs of the same length (11-eicosanoic acid, erucic acid and nervonic acid, respectively) were identified as contributing to biomass formation. All of these FAs have been reported in sponges previously (see references above). Eleven out of twenty-three FAs considered important for biomass production were unknown. Sponges, and their associated bacteria, have been shown to produce extensive modifications including methylation [28] and hydroxylation [33] that would prevent the matching of novel compounds to commercial FA databases. This diversity and high number of unknown compounds highlights the complexity of the *A. queenslandica* lipidome and the potential for novel compound discovery.

The FAME analysis also identified the sterol fraction of the extracted lipids. The three sterols that fit our criteria of contributing to the overall biomass were brassicasterol, cholesterol, and an unknown sterol (UU13). Surprisingly, the most abundant of these was the brassicasterol, a plant steroid hormone [35] that is synthesized by terrestrial and marine plants, including macro and unicellular algae. Brassicasterol has been previously reported in a range of filter feeding and grazing marine invertebrates including sponges [36], gorgonian corals (Cnidaria; Anthozoa; Octocorallia; Gorgonacea) [37] and sea urchins (Echinodermata; Echinozoa; Echinoidea) [38]. In these other classes of animals, brassicasterol is a minor sterol and its presence could be a result of dietary uptake. This is likely the case for the *A. queenslandica* holobiont, as neither animals nor bacteria have been shown to synthesize brassicasterol [36,37,38]. Nevertheless, the high abundance implies an important biological function that this study is unable to discern. While brassicasterol is an unusual, dominant sterol for an animal, cholesterol is considered an important component of all cellular membranes in animals [23]. The concentration of cholesterol in a membrane performs two important functions. The overall fluidity of a membrane is regulated by the amount of cholesterol. Increases in cholesterol content reduce the fluidity of the membrane and permability of neutral ions [39]. Membrane bound cholesterol can also act as a promoter or inhibitor of membrane bound protein reactions [40]. Additionally, cholesterol functions as an important substrate molecule for bioactive compounds and other sterols, including brassicasterol [35]. Without identifying the second most abundant sterol, UU13, we are unable to infer its biological role, or synthesis pathway.

### 3.3. Amino Acid Composition

Analysing the amino acid composition identifies the relative amounts of precursor molecules that are required to synthesis the protein component of the biomass. When complemented with information from genome scale modeling of the synthetic pathways that are present in an organism, these data allow for the identification of essential amino acids that cannot be produced by the organism [5]. Subsequent flux analysis can identify bottlenecks where the high requirement of a compound for biomass synthesis slows overall growth. The protein component of *A. queenslandica* comprises of two distinct products of metabolism. The overall sponge biomass consists of soluble, cellular derived protein, and an insoluble, extracellular protein matrix. This extracellular matrix is dominated by a sponge specific collagen called spongin [19], which is the main component of the sponge skeleton.

We defined the skeleton of *A. queenslandica* as the inorganic silica spicules, together with the organic collagen matrix. Collagen is an important structural component of metazoans. We found that the spongin in *A. queenslandica* contained 34.9% glycine (Table 6). This is close to the values reported for *Chondria reniformis* (30.5%) ([41], *Scypha graminae* (31.5%–32.3%) [42] and *Ircinia* sp. (32.3%) [43]. Alanine (10.3%) and proline (7.5%) were the next two most abundant amino acids (Table 6) and are very close in abundance to the values of these other sponges. Although the amino acid composition of sponge collagens is well documented [41,42,43], the amino acid composition of the complete sponge biomass has not been reported.

The complete amino acid composition may give insights into the many primary cell culture attempts that so far have had limited success [44]. Many studies have utilised mammalian cell culture media, with and without serum [44]. A comparison of the amino acid composition of the complete *A. queenslandica* biomass (Table 4) with a hybridoma cell line [45] revealed nine amino acids that are at least twice as abundant in *A. queenslandica*. Proline and glycine stand out at 3.3 and 8.5 times more abundant in *A. queenslandica* than the hybridoma line. If we use DMEM F12 medium as an example of mammalian cell culture media, both proline and glycine are minor contributors to the amino acid fraction [46] and could present significant bottlenecks for the production of biomass. In addition, due to the different amino acid bias in serum compared to sponge biomass, in the serum experiments excess waste amino acids could lead to growth limitations and may point to necessary changes in the approach to sponge cell culture.

We analysed the amino acid composition of the complete sponge biomass. From this we were able to estimate the average amino acid composition of the cellular fraction by subtracting the average composition of the skeleton. Values were then normalized for comparison by converting to a percentage. This showed that the cellular composition was distinct from the skeleton. The skeleton proportionally contained more glycine, alanine and proline than the cellular biomass. These three amino acids are key in giving collagen its triple helix shape and physical properties [23]. Amino acids that are essential in higher animals—in particular isoleucine, leucine, and valine [23]—were proportionally higher in abundance in the cellular biomass than in the skeleton. Whether these are also essential amino acids for *A. queenslandica* is yet to be shown.

## 4. Experimental Section

### 4.1. Sample Collection and Preparation

*A. queenslandica* were collected from Shark Bay, Heron Island, Australia (23°26′37.92″ S, 151°55′8.81″ E). Individual sponges and their coral rubble substrate were collected with a hammer and chisel [25] and transported submerged to the Heron Island Research Station. Sponges were held in a flow through aquaria system and processed within 8 h of collection. Biopsy samples were collected from a total of 29 sponges. The samples were cut with a sterile scalpel and contained a representative cross section of sponge biomass that consistently included the pinacoderm layer through to biomass just above the substratum. A thin layer of biomass was removed from the bottom of the sponge to avoid contamination by the substrata. Pieces of sponge biomass that had foreign material incorporated were avoided. To investigate the variation in composition within individual sponges, biopsies were collected from each sponge in multiples of 5. This resulted in 20 sponge samples per analysis, comprising 5 biopsy samples each of 4 individual adult sponges. Samples were snap frozen in liquid nitrogen then stored at −80 °C and transported at on dry ice. Lyophilisation was performed overnight. Dry weights were recorded on an analytical balance and corrected for salt content by multiplying the amount of water lost through lyophilisation by the specific gravity of seawater. We assumed that the salt content of the cells was the same as the seawater. Throughout the study, the weight of each reagent in each reaction was recorded. The specific gravity of each reagent was then used to ensure that accurate calculations were made.

### 4.2. Skeleton Dry Weight

Cellular material was removed from the *A. queenslandica* biopsies by re-hydrating with a 2% SDS solution made with MilliQ water and incubating for 2 h at room temperature on a shaker table. The biopsies were then washed thoroughly for 5 min with running RO water. The SDS incubation and washing times were optimized by inspecting with a Nikon Eclipse Ti-E inverted microscope (Nikon, Tokyo, Japan) to ensure all cellular material was removed. The pieces were then lyophilised overnight and dry weights recorded. These samples were stored at −80 °C.

### 4.3. Nucleic Acids

DNA was quantified by Hoechst fluorescence assay. The lyophilised samples from 4.1 were re-hydrated with 3.0 mL of TE buffer (pH 8.0) with 0.01 g/mL lysozyme and incubated at 37 °C for 1 h on a shaker table set to 100 rpm. Re-hydrating the lyophilised biopsies caused complete lysis of the *A. queenslandica* cells, but not the bacterial fraction. Lysozyme was added to ensure lysis of the bacterial fraction. After incubation, sterile tweezers were used to remove the sponge skeleton from the tube. The piece of skeleton was squeezed and rolled against the inside wall to ensure that the lysis buffer was retained. Samples were then vortexed and centrifuged for 5 min at 4000 g to pellet cellular debris. Aliquots of the supernatant were used in the Hoechst fluorescence assay. Calf thymus DNA was used to generate a standard curve [47]. All samples and standards were measured in triplicate.

RNA content was quantified by digesting the lyophilised samples from 4.1 with 3.0 mL of 0.3 M KOH for 1 h at 37 °C [48]. The reaction was then acidified with the addition of 1.0 mL of 3 M perchloric acid. [49]. Following centrifugation to remove DNA and protein, the RNA-containing supernatant was quantified in triplicate by UV absorbance at 260 nm [48].

The nucleotide composition of both the DNA and RNA was calculated using the GC content of the genome and an adult transcriptome. The calculations assumed that the DNA and RNA is all sponge derived as the bacterial genomic and transcriptomic sequence data, in addition to bacterial diversity and abundance data is not yet available. The GC content of the *A. queenslandica* genome and adult transcriptome are 31.14% [18] and 39.76% [20], respectively. The percentage contribution of each nucleotide was calculated, assuming an even guanine-cytosine and thymine-adenine/thymine-uracil ratios. Next the average molecular weight of one nucleotide was calculated. This was done by multiplying the abundance of each nucleotide by its molecular weight. These values were then added together to give the average molecular weight. This was multiplied by the mean total amount of DNA or RNA (g/gDW) to give the number of mols of average nucleotide per gram of dry weight. The amount of each nucleotide (mols) was then calculated using this value and the guanine-cytosine to thymine-adenine/thymine-uracil ratios. Values were converted to micromoles for reporting.

### 4.4. Protein

Sponge biopsies from 4.1 were digested with 3.0 mL of 1.0 M NaOH incubated at 95 °C for 5 min. The reaction was cooled in iced water and then centrifuged at 4000× *g* for 5 min to pellet suspended skeleton solids. The supernatant was assayed in triplicate using the Coomassie Plus™ (Bradford) Assay Kit (Thermo Scientific, Rockford, MI, USA). Bovine serum albumin was used as the standard [50].

### 4.5. Carbohydrate

Carbohydrate content was quantified using the phenol-sulfuric acid method [51]. The sponge biopsies from 4.1 were rehydrated with 1.0 mL of MilliQ water. 5.0 mL of concentrated sulfuric acid was added and the reaction let proceed for 10 min. The reaction was rapidly cooled by placing the tube in iced water for 1 min. [52]. 50 μL of phenol was added and incubated for 30 min in a water bath set to 25 °C. The reaction was diluted 1:50 using concentrated sulfuric acid and the absorbance read at 488 nm. All measurements were taken in triplicate and glucose was used as the standard [52].

### 4.6. Lipid

Biopsy samples from 4.1 were rehydrated with 1.0 mL MilliQ water and extracted overnight with 3:2 hexane:isopropanol [53]. 5.0 mL of 0.47 M NaSO_4_ was added, mixed and then separated by centrifugation at 4000× *g* for 5 min. The hydrophobic upper hexane phase contains the lipid fraction and was transferred to a clean glass tube. The initial solution was washed with 7:2 hexane:isopropanol to ensure that all of the lipids were recovered [53]. This second hexane phase was again recovered with centrifugation and combined with the first extract. The combined hexane extracts were evaporated under a constant flow of nitrogen to prevent oxidation. The remaining solid lipids were quantified gravimetrically. The lipid solid was then stored under nitrogen at −80 °C for fatty acid analysis.

### 4.7. Fatty Acids and Sterols

A fatty acid methyl ester (FAME) analysis was performed on the extracted lipids as previously described [54]. The lipid solid was dissolved in hexane and 5.1 μg of nondecanoic acid (Sigma-Aldrich, St. Louis, MO, USA) was used as an internal standard (ISTD) to a final concentration of 3.6 μg/mL. Saponification, methylation and GC/MS analysis of the lipid mass fraction followed the protocol described in [54] The software program AMDIS (version 2.64, National Institute of Standards and Technology (NIST), Gaithersburg, MD, USA) was used for the analysis of the fatty acid and sterols. A minimum match factor of 80 and signal threshold of 700 was set for identifying compounds. A FAME library was built using the target ions and retention times of thirty-two known fatty acid standards (Sigma-Aldrich, St. Louis, MO, USA). An alkane (C_7-30_) standard was used as a retention time index. To define compounds important for biomass formation, we used a cut-off of 0.05% of the largest peak in the MS analysis, which means that peaks below those signal intensities would not be reported. For unknown compounds (*i.e.*, that were not identified using the standard library), their fragmentation patterns were compared to the NIST database library (version 2.0, NIST, Gaithersburg, MD, USA). For a range of peaks both the target library as well as the NIST library did not provide accurate hits. Based on manual interpretation of fragmentation patterns, unknowns that were found consistently in the majority (>75%) of the samples were classified into sterols or fatty acids. From the quantified lipid fraction (g/gDW) the estimated weight of glycerol and phosphate groups was subtracted assuming a glycerolipid to phospholipid ratio of 20:80. It was then assumed that the reminder of the lipid fraction would generate a signal in GC-MS and that the MS response and ionization efficiency for all FA and sterol ions was comparable (due to the unknown character of many FA’s this cannot be tested, but individual errors for individual compounds will not impact dramatically on the later modelling). All peak areas were normalized to the total signal generated for that sample. Using the molecular weight of the knowns and an average molecular weight (calculated from all identified FA’s and sterols) for the unknowns the individual concentrations were calculated in mol/gDW. A total of 23 separate fractions of biomass were analyzed and the standard deviation was calculated.

### 4.8. Amino Acids

Amino acid analysis was performed by reversed phase high performance liquid chromatography. 20 samples containing cellular and skeleton components were analysed in addition to 20 samples of skeleton, prepared as above. 5 mL of 6 M HCl was added to each sample and incubated at 105 °C. Hydrolysis times were first optimized and found to be 54 h for the complete biomass samples and 48 h for skeleton samples. Optimization monitored the concentration increase of all amino acids over the time course and 54/48 h was found to be a time at which most amino acids reached the peak concentration. Longer times may favor some compounds but will lead to degradation of others. This compromise was necessary since otherwise individual experiments need to be conducted for all 20 amino acids. Samples were allowed to cool, mixed, and then a 500 μL aliquot was taken and filtered with a 0.22 μm Ultrafree-MC GV centrifugal filter (Merck Millipore, Darmstadt, Germany). The HCl was evaporated by heating to 40 °C under a constant flow of nitrogen. The solid pellet was stored at −80 °C. Derivatisation was performed in a high-performance autosampler (Agilent HiP-ALS SL, G1367C, Santa Clara, CA, USA). 0.5 μL of sample containing 250 μM of internal standards, sarcosine and 2-aminobutyric acid, was added into 2.5 μL of borate buffer (0.4 N, pH 10.2, Agilent PN: 5061-3339, Santa Clara, CA, USA), mixed and incubated for 20 s at 4 °C. 1 μL of OPA reagent (10 mg *o*-pthalaldehyde/mL in 3-mercaptopropionic acid, Agilent PN: 5061-3335, Santa Clara, CA, USA) was then added to initially derivatise primary amino acids. The reaction was mixed and incubated for 20 s at 4 °C. Then 0.4 μL of FMOC reagent (2.5 mg 9-fluorenylmethyl chloroformate/mL in acetonitrile, Agilent PN:5061-3337, Santa Clara, CA, USA) was added, mixed and incubated for 20 s at 4 °C to derivatise the secondary amines proline and sarcosine. 45.6 μL of Buffer A (40 mM Na_2_HPO_4_, 0.02% NaN_3_, pH 7.8) was added to lower the pH of the reaction prior to injecting the 50 μL reaction onto an Agilent Zorbax Extend C-18 column (3.5 μm, 4.6 × 150 mm, Agilent PN: 763953-902, Santa Clara, CA, USA) with a guard column (SecurityGuard Gemini C18, Phenomenex PN: AJO-7597, Santa Clara, CA, USA). Column temperature was kept at 39 °C in a thermostatted column compartment (Agilent TCC, G1316B, Santa Clara, CA, USA). Chromatography was performed using an Agilent 1200-SL HPLC system, equipped with an active seal wash and a degasser (Agilent Degasser, G1379B, Santa Clara, CA, USA). The HPLC gradient was 2%–45% buffer B (45% acetonitrile, 45% methanol and 10% water) from 0 to 18 min, 50%–60% buffer B from 18.1 to 20 min. 100% buffer B from 20.1 to 24 min, and 2% buffer B from 24.1 to 27 min—using a binary pump (Agilent Bin Pump SL, G1312B, Santa Clara, CA, USA). Flow rate was 2 mL/min. Derivatised amino acids were monitored using a fluorescence detector (Agilent FLD, G1321A, Santa Clara, CA, USA). OPA-derivatised amino acids were detected at 340_ex_ and 450_em_ nm from 1 to 18 min, and FMOC-derivatised amino acids at 266_ex_ and 305_em_ nm from 18 to 27 min. Chromatograms were integrated using ChemStation (Rev B.03.02, Agilent, Santa Clara, CA, USA).

### 4.9. Statistical Analysis

To analyse how the variation between and within individual sponges contributed to the overall variation for each macromolecule separately, the data were fit to a linear mixed effects model using the *nlme* package [55] in R [56].

The amount of macromolecule (g/gDW) was the response variable and Sponge ID (individual) was the random effect. The among-individual variance was divided by the total variance and expressed as the percentage of the overall variability that was due to variation between individuals. To address whether this between-individual variation was significant, we used a likelihood ratio *x*^2^ test to compare two models: one with the random effect and one without the random effect.

## 5. Conclusions

As a prerequisite for a systems scale investigation of the metabolic processes responsible for sponge biomass production, we analysed the composition and abundance of the macro-components of *A. queenslandica.* We found the composition to be within the reported ranges for other tropical marine sponges and that there is significant variation in the composition at the population level and notable variation within the biomass of an individual sponge. The skeleton was the most abundant component of the overall biomass, while lipids and protein were the most abundant macromolecules. Further analysis of the fatty acid and amino acids was undertaken. The FAME analysis revealed a diverse and complex fatty acid composition. The composition of the sterols revealed that brassicasterol and an unknown sterol had a higher mean abundance than cholesterol. There was a distinct difference in amino acid composition between the soluble, cellular fraction and the insoluble skeletal component. This highlights the different protein products of metabolism that share the same pool of precursor molecules. This complete analysis of the biochemical composition of *A. queenslandica* provides baseline information that complements the extensive genomic resources available for *A.* *queenslandica*, a key species for understanding sponge biochemistry and metabolism.
